# Occupational Exposure during Pregnancy and Effects on Newborns: A Nested Case-Control Study

**DOI:** 10.3390/life13101962

**Published:** 2023-09-26

**Authors:** Gabriele Donzelli, Beatriz Marcos-Puig, Isabel Peraita-Costa, Juan Llopis-Morales, María Morales-Suarez-Varela

**Affiliations:** 1 Institute of Clinical Physiology of the National Research Council (CNR-IFC), 56124 Pisa, Italy; gabriele.donzelli@ifc.cnr.it; 2 Department of Health Sciences, University of Florence, 50134 Florence, Italy; 3 Department of Gynecology and Obstetrics, La Fé University and Polytechnic Hospital, Avda. Fernando Abril Martorell 106, 46026 Valencia, Spain; maria.b.marcos@uv.es; 4 Research Group in Social and Nutritional Epidemiology, Pharmacoepidemiology and Public Health, Department of Preventive Medicine and Public Health, Food Sciences, Toxicology and Forensic Medicine, Faculty of Pharmacy, Universitat de València, Av. Vicent Andrés Estelles s/n, Burjassot, 46100 Valencia, Spain; isabel.peraita@uv.es; 5 Biomedical Research Center in Epidemiology and Public Health Network (CIBERESP), Carlos III Health Institute, Av. Monforte de Lemos 3-5 Pabellón 11 Planta 0, 28029 Madrid, Spain; 6 Faculty of Pharmacy, Universidad Alfonso X el Sabio, Avda. de la Universidad 1, Villanueva de la Cañada, 28691 Madrid, Spain; juanllopis6@gmail.com

**Keywords:** women’s occupational exposures, chemical risks, biological risks, physical risks, reproductive health

## Abstract

Background: The protection of pregnant workers should be based on evidence regarding the risks to reproductive health from exposure to specific work environments and conditions. The objective of this study was to identify the effects on mothers and newborns resulting from environmental exposure to various occupational risks. Methods: The study cohort was composed of 399 women admitted to the Obstetrics/Postpartum ward at Hospital La Fe in Valencia, Spain. Face-to-face interviews were conducted to establish associations between workplace exposure during pregnancy and its effects on maternal and newborn health. Sex, anthropometric characteristics, and blood gas analysis in arterial and venous umbilical cord blood at delivery were collected. Results: A total of 138 women were exposed to biological and/or chemical risks, 122 to physical risks, and 139 at no risk of exposure. In the group with chemical and/or biological risks, the frequency of women who resorted to in vitro fertilization to achieve the studied pregnancy is less than half of the group exposed to physical risks, with statistically significant differences (*p* = 0.047). The mean values for the arterial analysis in both exposure groups were within average values, with similar pH values between them, but the mean values of PCO2 and PO2 were lower in the group of neonates of mothers exposed to physical risks, with a significant difference for arterial PO2 (*p* = 0.027). Conclusion: Our analysis contributes evidence for planning and prioritizing preventive actions to protect women’s reproductive health. The results suggest the continuation of a future project that would consider more factors and potentially increase the sample size.

## 1. Introduction

The protection of pregnant workers should be based on evidence regarding the risks to reproductive health from exposure to specific work environments and conditions. Several studies have shown that certain physical hazards in the workplace, such as ionizing radiation, electromagnetic fields, and noise, as well as exposure to chemical agents such as lead, mercury, cadmium, solvents, pesticides, ethylene oxide, anesthetic gases, and pharmaceuticals, have negative consequences for women’s reproductive health in terms of fertility, risk of fetal loss, and pregnancy outcomes, as well as on the newborn, when it comes to anthropometry, APGAR scores, and blood gas levels at birth [1,2,3,4,5,6,7,8,9,10,11,12,13,14,15].

Moreover, the physical demands of work, such as lifting heavy loads or prolonged standing, have also been linked to adverse effects on pregnancy and fetal development [16]. Psychosocial factors such as shift and night work, stress, job dissatisfaction, and excessively long working hours have demonstrated implications for reproductive health [17]. Additionally, risks associated with exposure to biological agents, including rubella, cytomegalovirus, hepatitis virus, and human immunodeficiency virus, are well known [18], and these exposures have been linked to adverse outcomes, such as spontaneous abortion, low birth weight, preterm births, syndromes, congenital malformations, central nervous system defects, and delayed psychomotor development [19,20,21,22,23,24].

Fetuses and children are particularly vulnerable to environmental toxins due to their physiological immaturity and extended exposure time following exposure. In recent years, there has been increased interest in studying the effects of environmental exposures on fetal health. The developmental effects can have immediate and long-term consequences for individuals’ health. Until relatively recently, there were only a few studies on the impact of prenatal exposure to environmental pollution on fetal and neonatal health [25].

Low birth weight and preterm birth are significant predictors of perinatal morbidity and mortality [26]. Due to their clinical relevance and profound social and economic implications, they are critical indicators for public health [27]. Preterm and low-birth-weight children are more likely to die during the first months or years of life [28]. Additionally, they are more prone to childhood illnesses [29], developmental difficulties, and even health issues in adulthood. Hence, it is essential to identify factors related to these problems to develop necessary preventive measures. Some determinants associated with this issue are linked to maternal employment and occupation during pregnancy [30]. Early studies conducted in the 1950s and 1960s suggested that maternal work posed a risk to pregnancy outcomes [31,32,33,34]. However, subsequent research has found better pregnancy and birth outcomes in working women.

Therefore, it seems pertinent to question whether having a job outside the home during pregnancy can affect the fetus, newborn, or the duration of gestation and potential effects during the gestation period. It is important to determine if the differences in the risk of low birth weight or preterm births, newborn birth problems, and maternal health effects are related to maternal occupation, especially considering the increase in women’s labor force participation in most European Union countries. This is in light of the fact that the employment rate for women (aged between 20 and 64) in the European Union (EU) stood at 67% in 2018, a 1 percentage point (pp) increase from the previous year and 5 pp higher than in 2008 [35].

The objective of this study was to identify the effects on mothers and newborns resulting from occupational exposure to various occupational risks.

## 2. Materials and Methods

The design of this study is nonexperimental and observational, consisting of two phases: a descriptive phase to characterize the studied sample and an analytical phase with a nested case-control design on a retrospective cohort.

In our study, the cohort is composed of women who, after childbirth, were admitted to the Obstetrics/Postpartum ward at Hospital La Fe in Valencia. To establish a potential association between occupational exposure during pregnancy and its effects on maternal and newborn health, we considered the different chemical, biological, and physical risks in the mother’s work environment during pregnancy as the exposure factor. The possible outcomes of this exposure on maternal and newborn health were considered as the effects. The effects on newborn anthropometry, APGAR scores, and blood gas levels were evaluated at the time of birth, while the maternal occupational exposure and other characteristics, including maternal health measures, such as fertility, risk of fetal loss, and pregnancy outcomes, were collected at the time of birth but assessed retrospectively.

For the descriptive part of our study, we analyzed parental characteristics (personal, socioeconomic, and anthropometric); maternal clinical and obstetric history; and data related to gestation and childbirth, including the newborn’s condition, anthropometry, APGAR scores, and blood gas levels. In the analytical part of the study, these data were related to maternal occupational exposure based on the occupation during pregnancy and its potential effects on maternal and newborn health.

Based on the different environmental workplace risks as the exposure variable, we identified cases as mothers with exposure to chemical and/or biological risks and mothers with exposure to physical risks. Controls were pregnant women without workplace exposure. The information for these three groups was obtained in parallel and randomly, ensuring the homogeneity and representativeness of the study population.

### 2.1. Study Population

The target population for this study includes pregnant mothers who gave birth at Hospital La Fe in Valencia between February 2016 and June 2016 and were admitted to the Obstetrics/Postpartum ward for 2 to 3 days, as per the established protocol. The population includes both the mothers and their respective newborns, who may be admitted to the same ward, Neonatology (NN) or the Neonatal Intensive Care Unit (NICU).

**Figure 1:**
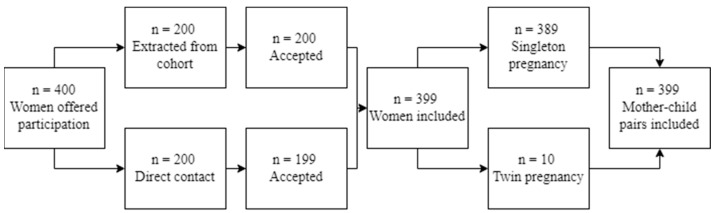
Participant selection flow diagram.

The study included mother–child pairs who were admitted to the Obstetrics/Postpartum ward after childbirth and agreed to participate in the study. Out of 400 mothers who were offered participation, 200 were contacted directly during their postpartum hospital stay, and the remaining 200 were randomly selected and extracted from the database registry of the Department of Preventive Medicine and Public Health at the Faculty of Pharmacy. The participation rate in data collection was 99.5% for the mothers contacted in person (n = 199) and 100% for the cohort (n = 200) from the Department of Preventive Medicine and Public Health. A total of 399 mothers were included in the study. Out of these, 389 women hade singleton pregnancies, and 10 had twin pregnancies (Figure 1). For the purposes of this study, only one baby from each twin pregnancy was considered. Thus, the final number of mother–child pairs included in the study was 399 (n = 399).

Inclusion criteria for the study population required the consent of pregnant mothers to participate in the study and the availability of newborn data. Exclusion criteria encompassed mothers who did not provide consent, lack of available data on the newborn’s characteristics, incomplete or inconsistent responses from mothers, maternal admission to the Intensive Care Unit after delivery, stillbirth, or neonatal death shortly after birth.

Mothers included in the study signed a consent form evaluated and approved by the Ethics Committee of Clinical Research at Hospital La Fe in Valencia, ensuring the confidentiality of collected data according to the Organic Law 15/13 December 1999 on Personal Data Protection.

### 2.2. Data Collection

The data for this study were collected through face-to-face interviews with mothers during their postpartum admission at the Obstetrics/Postpartum ward at Hospital La Fe in Valencia. Additionally, relevant information was gathered from the medical records of both mothers and newborns.

The interview was structured using a questionnaire with various sections dedicated to obtaining all the necessary information for the study. The data collected were both qualitative and quantitative, and this was considered during the coding and statistical analysis. For digitization, different variables were encoded using a binary numerical system (0/1) for qualitative dichotomous variables and the decimal numbering system for qualitative nominal and quantitative variables. All the information was entered into an Excel database using Microsoft Excel 2010.

### 2.3. Assessment of Employment Status and the Level of Physical Activity

The questionnaire included a first section comprising three questions to assess the maternal employment status during pregnancy. The first question determined employment status, whether the mother was employed or unemployed at the time of birth and during pregnancy. The second question inquired about the type of job the mother had carried out during pregnancy. The third question gathered information about the level of physical activity during pregnancy in the workplace (sedentary behavior, light physical efforts, or intense physical efforts and movements). Sedentary behavior was defined as sitting most of the day/standing for most of the day, without much movement or effort; light physical activity, such as walking, carrying some weight, and making frequent journeys; and intense physical activity, such as performing tasks that require great physical effort.

Based on the maternal occupational information obtained in the survey, a classification was made according to previously conducted studies [36], using the International Standard Classification of Occupations (ISCO) and Nomenclature of Economic Activities (NACE) to determine the potential risks associated with the occupations of the studied mothers. After the classification of the maternal occupations and their associated risk, women were categorized into one of three exposure groups: no exposure (unemployed mothers), chemical/biological risk (mothers exposed to biological and/or chemical risks), and physical risk (mothers exposed to physical risks).

### 2.4. Analysis

The maternal workplace exposure was categorized into three groups: no exposure, chemical/biological risk, and physical risk. Descriptive statistics were used to analyze the study population in each category. For quantitative variables, the mean and standard deviation were determined, while for qualitative variables, the frequency or percentage in the study population was studied. ANOVA was used for comparing different variables in maternal occupational exposure categories for quantitative variables, and chi-square test was used for qualitative variables. The level of significance was set at *p* < 0.05. The IBM SPSS 28.01.01 statistical software was used for analysis.

## 3. Results

Out of the 399 women included in this study, 138 (34.6%) were exposed to biological and/or chemical risks in their workplace environment, compared to 122 women (30.6%) exposed to physical risks in the same environment. Table 1 shows the distribution of occupational categories during pregnancy based on different workplace environmental risk groups. Regarding the ISCO, occupations with physical risks predominated compared to those with no exposure or with chemical and/or biological risks, and this difference was statistically significant (*p* = 0.047). On the other hand, the volume of economic activities with chemical and/or biological risks in the NACE is noteworthy, but no statistical differences were found.

**Figure 2:**
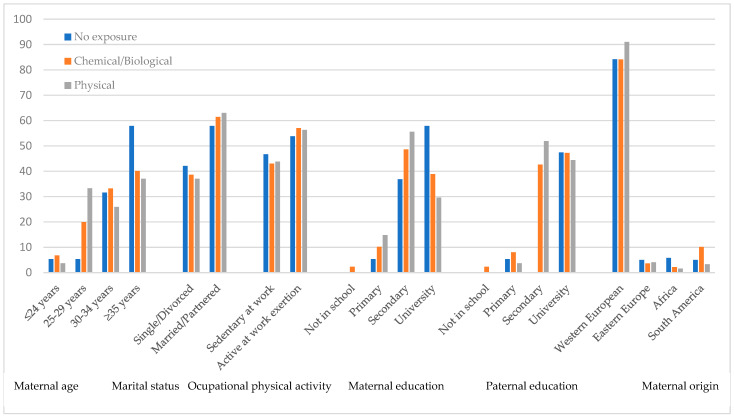
Personal and sociodemographic characteristics.

Pregnant women in the younger age group, aged 24 years or younger, had lower occupational exposure, with fewer potential resulting risks, with a maximum percentage of 6.8% in the group with biological and/or chemical risks (Figure 2). However, it is notable that pregnant women over 35 years old had a higher exposure to these same risks in their workplace, with a percentage of 40.1%. Nevertheless, no statistically significant differences were found (*p* = 0.341).

Similarly, in the group of women exposed to biological and/or chemical risks during pregnancy, a higher proportion resided in a family unit, had a higher level of education for both the mother and father, and had a higher frequency of having Western European nationality.

Regarding marital status, 61.4% of women in the group with biological and/or chemical risks, 63.0% in the group with physical risks, and 57.9% in the group with no exposure declared being married or in a partnership. The percentages of pregnant women in these marital statuses were higher compared to the percentages of single or divorced women, but these differences were not statistically significant (*p* = 0.939).

Regarding the level of physical activity at work, in the group with chemical and/or biological risks, 43.0% had sedentary behavior at work, and 57.0% had movements or engaged in light to moderate physical efforts during their workday. In contrast, in the group with physical risks, a higher percentage (56.3%) of pregnant women performed light-to-moderate movements/efforts compared to those with sedentary behavior in their work routine.

However, statistically significant differences were found in relation to the mother’s level of education for the studied groups of workplace exposure (*p* = 0.019). For the group exposed to biological and/or chemical risks, 42.6% had secondary education, and 47.2% had a university education; meanwhile, only 2.3% of mothers reported no schooling, and 8.0% had primary education. For the group exposed to physical risks, it is worth noting that 51.9% had secondary education and 44.4% had a university education, while none of the mothers exposed to these risks reported no schooling, and only 3.7% had primary education.

Regarding the country of maternal origin, no statistically significant differences were found. It is noteworthy that both in the group with biological and/or chemical risks and in the group with physical exposure, as well as in the group with no exposure, a higher frequency of women from Western Europe was observed (84.1%, 91.0%, and 84.2%, respectively).

As can be seen from the obstetric data of the mothers in Table 2, in the group with biological and/or chemical risks, there was a higher mean of multiparous women (1.10 ± 1.13), but also a higher mean number of abortions, which was 0.52 ± 0.77. In contrast, as expected, the lowest mean number of previous fetal loss was found in women without exposures during pregnancy (0.44 ± 0.83). It is important to note that statistically significant differences were found (*p* = 0.025) in the mean number of previous births, with a value of 0.82 ± 0.87 in pregnant women without exposure, 0.68 ± 0.79 in women with chemical and/or biological exposures, and 0.53 ± 0.713 in mothers with physical risks.

Regarding the characteristics of the pregnancy studied, a statistically significant difference was found for in vitro fertilization treatment (Table 3). On the other hand, no statistically significant differences were found for weeks of gestation and hospitalization during pregnancy.

Regarding the sex and anthropometric characteristics of the newborns in relation to occupational exposure (Table 4), in the group with chemical and/or biological risks, there were more male infants, i.e., 52.2%, compared to 47.8% female neonates. Similarly, in the group of women with physical risks, male births predominated, with 52.5% male and 47.5% female, showing no statistically significant difference between groups. The mean weight in the group of mothers with criteria for chemical and/or biological risks was 3201.9 ± 594.9 g, slightly higher than the mean weight of newborns of mothers with exposure to physical risks, where we found a mean of 3181.3 ± 534.2 g; however, this difference was not statistically significant (*p* = 0.915). The mean height was also slightly lower in the latter group, while the mean head circumference was slightly higher, but not significant in either case. Regarding the Apgar test results, both in the first and fifth minute, the mean score was lower in the newborns of the group of women with chemical and/or biological exposure, with statistically significant differences in the first minute.

In Table 5, the values of the arterial and venous blood gas analysis of the umbilical cord are presented. First, the arterial analysis shows that the mean pH value was quite similar in neonates from both groups, while the mean values of both PCO2 and PO2 were lower in the group of neonates of mothers with physical risks in their workplace environment. Here, it is worth noting the difference in means for PO2 between both groups, as it is statistically significant, with a mean of 21.96 ± 10.18 mmHg in the group with chemical and/or biological risks and 20.46 ± 8.70 mmHg in the group with physical risks, with a significance level of *p* = 0.027.

Second, the venous analysis of cord blood gases is shown. In this case, similarly to the previous case, no significant differences were found in pH. Likewise, a higher mean PO2 was found in neonates of the group with chemical and/or biological risks; however, no statistically significant differences were found.

Regarding the factors considered in the newborn in Table 6, an increase in the odds ratio (OR) was found for the relationship between physical risk and arterial PO2 value (adjusted OR, 1.96; 95% CI, [0.88–4.08]). This increase was obtained when adjusting the relationship with variables such as gestational week at birth, maternal age, and physical exercise at work. On the other hand, for the relationship between workplace exposure and Apgar score at one minute, an increase was observed in the group with chemical and/or biological risks when adjusting with variables such as gestational week at birth, maternal age, and physical exercise at work (adjusted OR, 1.35; 95% CI, [0.31–5.85]).

## 4. Discussion

In the present study, it was identified that more than half of the women were exposed to work-related risks (65.2%), with 34.6% of women exposed to chemical and/or biological risks in their workplace and 30.6% of them exposed to physical risks. Additionally, the frequency of exposure to these risks was related to sociodemographic characteristics, including maternal age, mother’s and father’s educational level, and nationality, but differences among the exposure groups only appear for maternal educational level.

As previously mentioned, all considered risk categories have some potential negative effects on pregnancy. However, our data do not allow us to estimate the intensity of exposure to these factors. Further evaluations would be necessary to calculate the magnitude of these exposures and determine the intervention needs in each case.

Sociodemographic and maternal lifestyle characteristics: One notable maternal factor was maternal age in relation to work exposure, where the percentage of women aged 35 or older exposed to chemical and/or biological risks was 40.1%, and that of the group exposed to physical risks in the studied sample was 30.7%. These results align with previous studies [37]. This could indicate a current trend in society towards delayed pregnancies, possibly due to changes in lifestyle and socioeconomic situations, leading women to become mothers later in life. There is a global trend to postpone motherhood, which is more pronounced in developed countries. Late pregnancies, after the age of 35, need to be closely monitored due to the inherent risks associated with such pregnancies, which may interact synergistically with occupational exposures [38].

In the same vein, the educational level of the pregnant woman was also evaluated, and statistically significant differences were found (*p* = 0.019). For the group exposed to biological and/or chemical risks, 42.6% of pregnant women had secondary education, and 47.2% had university education, compared to 2.3% of mothers who reported no schooling and 8.0% with primary education. For the group exposed to physical risks, it was notable that 51.9% had secondary education, and 44.4% of pregnant mothers had university education, compared to none of the mothers declaring no schooling and 3.7% declaring only primary education.

No statistically significant differences were found between the maternal country of origin and occupational exposure, probably due to the minor representation of foreign participants in the study. Similar results were not found significant when evaluating the same variable in similar studies [39].

For this study, it was crucial to assess the maternal employment status. One of the factors considered for this evaluation was the physical activity of the pregnant woman during her working hours. Although statistical significance was not obtained in the present analysis, there was a prevailing trend among mothers who engaged in light-to-moderate physical exertion or movements during their work. Excessive or inappropriate physical activity during pregnancy increases energy expenditure and negatively impacts fetal nutrition. Excess physical load can retard fetal growth through its effect on blood flow since both exercise and certain work postures reduce uteroplacental blood flow [40]. However, other authors have found a significant association between physical work, prolonged standing, fatigue, and lifting heavy weights at work with preterm birth [41]. Environmental conditions in the workplace, such as noise, hot ambient temperature, and humidity, can also influence the final outcome of pregnancy [42].

During the second and third trimesters of pregnancy, the fetus requires a continuous supply of nutrients for its normal development and needs to avoid exposure to toxins that hinder its normal growth. In this regard, different maternal occupational exposures to certain chemicals, such as solvents or metals, among others, can reach the fetus through the placenta and hinder its development, leading to low birth weight [43].

There is significant controversy in the literature on this topic, manifesting the difficulties in answering the question of how the type of work performed by the mother during pregnancy affects the duration of gestation and birth weight [44].

The mean scores for the Apgar test in the first minute were statistically significant (*p* = 0.047), being slightly lower in the chemical and/or biological risk group. There seem to be no studies that relate Apgar test results to occupational exposure during pregnancy [37]. As a simple scoring method, it is routinely used in neonatal evaluation immediately after birth. The score at one minute of life serves as a guide for resuscitation, and the score at five minutes indicates the effectiveness of resuscitation or provides an idea of extrauterine adaptation [45]; however, it cannot be interpreted in isolation for the diagnosis of perinatal asphyxia [46].

Data from umbilical cord blood gasometry, which provides an objective measurement of the fetal condition before birth [47], were also collected. On the one hand, the arterial analysis reflects the fetal oxygenation and acid–base status. This blood has reduced oxygen and nutrient content and increased CO_2_ [48]. In the present study, the mean values for the arterial analysis in both exposure groups were within average values, with similar pH values between them, but mean values of PCO2 and PO2 were lower in the group of neonates of mothers exposed to physical risks, with significant difference for arterial PO2 (*p* = 0.027). The plasma partial pressure of oxygen is a good indicator of oxygenation status, but due to the characteristics of fetal hemoglobin, its variations and consequences in the fetus have been difficult to interpret [49]. On the other hand, the venous analysis reflects maternal oxygenation and acid–base status, as well as placental status, which, when interpreted along with arterial values, can provide an idea of the possible origin of fetal distress [48]. No studies were found to contrast these results of cord blood gasometry, perhaps because this practice is usually reserved for cases where adverse outcomes for the newborn may be expected due to intrapartum events [47].

However, intrapartum fetal asphyxia is a significant perinatal complication, diagnosed based on the pH, the Apgar index, neurological manifestations (hypotonia, seizures, or coma), and the dysfunction of two or more organs [50,51]. Hence, it may be interesting to consider these factors when planning new studies.

One of the strengths of this study was its execution on a population of pregnant women from the University and Polytechnic Hospital La Fe in Valencia, following homogeneous selection criteria. Likewise, all data were collected by the same interviewer in a personal and direct manner with the mothers, subsequently cross-referencing them with the clinical records of the mother and the newborn. Another strong point of this work is that it used the type of exposure in the workplace during pregnancy, with standardized work coding [37].

Regarding the limitations, it should be noted that the achieved sample size was not very large, and this limited the study. Therefore, in future investigations, increasing the sample size would be beneficial to obtain more robust results. Additionally, it should be mentioned that the information was collected retrospectively, which may lead to memory bias, possibly reinforced by a reluctance to admit unhealthy practices during pregnancy. The exclusion of women admitted to the Intensive Care Unit after delivery or who had suffered a stillbirth or neonatal death shortly after birth may have led to an underestimation of the effects of occupational exposure.

Another limitation stems from the fact that information about occupational exposures is based on self-reports from the workers, without access to other objective measurements, making it impossible to estimate whether the exposures they experienced exceeded any normative or reference limits. However, interviews are the only feasible approach to understanding the working conditions in the general Spanish population, as there is no population-based registry of occupational exposures.

In addition, it would be important to evaluate, in more detail, the implications of individual specific exposures, especially within the chemical exposure category, given the possible differences in their effects depending on the classes or types of chemicals to which pregnant women may be exposed.

## 5. Conclusions

The present study provides valuable information on the frequency of exposure to different biological, chemical, and physical risks and their potential effects on pregnancy and newborns. The prevalence of chemical, biological, or physical occupational exposures in pregnant women is notable. In this study, an increased risk of adverse effects was observed, although not statistically significant, as in the previously available literature. This would support the need for specific occupational prevention programs for pregnant women and/or women of childbearing age.

It is evident that to establish specific links between occupational exposures and reproductive and developmental problems, much more detailed information is necessary. Nevertheless, our analysis already contributes useful evidence for planning and prioritizing preventive actions to protect women’s reproductive health. The results of our study suggest the continuation of a future project, considering more factors and potentially increasing the sample size.

## Figures and Tables

**Table 1:** ISCO and NACE occupational classification according to the different risks in the work environment during pregnancy of the mothers studied.

	No Exposure	Chemical/Biological Hazard	Physical Hazard	*p*-Value *
	N = 139 Coding of Occupations	N = 138 Coding of Occupations	N = 122 Coding of Occupations	
**ISCO** **(2008)**	0	14, 21, 22, 23, 26, 31, 32, 34, 42, 51, 52, 53, 61, 72, 75, 91, 93	11, 12, 14, 21, 22, 24, 25, 26, 31, 33, 34, 35, 41, 42, 43, 44, 52, 73, 74, 75, 83	**0.047**
**NACE** **Rev.2**	4639, 4799, 6832, 7111, 7410, 7419, 7820, 8211, 8532, 8690, 8891, 8899, 9602, 9700	0161, 2016, 2592, 2892, 3109, 4299, 4639, 4719, 4724, 4762, 4799, 4941, 5210, 5610, 5629, 5819, 5829, 5920, 6203, 6391, 6411, 6832, 6910, 6920, 7010, 7022, 7111, 7172, 7312, 7320, 7410, 7430, 7490, 7500, 7820, 7911, 7912, 7990, 8129, 8211, 8411, 8413, 8510, 8520, 8532, 8610, 8621, 8623, 8690, 8731, 8891, 8899, 9602, 9620, 9700, 9820	0990, 4639, 5610, 6832, 7120, 7490, 8119, 8211, 8520, 9620	0.386

* *p*-value obtained by X^2^ test (*p* < 0.05).

**Table 2:** Median maternal obstetric history as a function of occupational exposure.

	No Exposure	Chemical/Biological Risk	Physical Risk	*p*-Value ^2^
	Mean ± SD ^1^	Mean ± SD ^1^	Mean ± SD ^1^	
**Previous pregnancies**	1.07 ± 1.09	1.10 ± 1.13	0.84 ± 0.965	0.101
**Previous births**	0.81 ± 0.87	0.68 ± 0.79	0.53 ± 0.713	**0.025**
**Previous fetal loss**	0.44 ± 0.83	0.52 ± 0.77	0.46 ± 0.855	0.692

^1^ Values correspond to the mean and standard deviation; ^2^ *p*-value obtained by ANOVA (*p* < 0.05).

**Table 3:** Characteristics of the pregnancy studied (N = 399) as a function of occupational exposure.

	No Exposure		Chemical/Biological Risk		Physical Risk		*p*-Value ^2^
	Mean o fr ^1^	±SD o % ^1^	Mean o fr ^1^	±SD o % ^1^	Mean o fr ^1^	±SD o % ^1^	
**In vitro fertilization**							
No	130	94.9	132	95.7	108	88.5	**0.047**
Yes	7	5.1	6	4.3	14	11.5	
**Weeks of gestation**	38.73	±1.56	38.84	±2.07	38.67	±1.96	0.765
**Hospitalization during pregnancy**							
No	121	92.4	130	95.6	114	95.8	0.395
Yes	10	7.6	6	4.4	5	4.2	

^1^ Values correspond to the mean and standard deviation for quantitative variables and to the number and frequency for qualitative variables; ^2^ *p*-value obtained by ANOVA (*p* < 0.05) for quantitative variables and by X^2^ test (*p* < 0.05) for qualitative variables.

**Table 4:** Sex and anthropometric characteristics of the newborn as a function of occupational exposure.

	No Exposure		Chemical/Biological Risk		Physical Risk		*p*-Value ^2^
	Mean o fr ^1^	±SD o % ^1^	Mean o fr ^1^	±SD o % ^1^	Mean o fr ^1^	±SD o % ^1^	
**Sex of newborn**							
**Male**	74	53.6	72	52.2	64	52.5	0.968
**Female**	64	46.4	66	47.8	58	47.5	
**Height of newborn (cm)**	49.7	±2.39	50.0	±2.50	49.2	±2.62	0.415
**Weight of newborn (g)**	3174.3	±555.8	3201.9	±594.9	3181.3	±534.2	0.915
**Head c Head circumference of newborn (cm)**	34.0	±2.54	33.9	±1.69	34.1	±2.32	0.791
**APGAR 1 min**	9.2	±1.18	8.9	±1.36	9.3	±0.83	**0.047**
**APGAR 5 min**	9.9	±0.34	9.8	±0.41	9.9	±0.34	0.224

^1^ Values correspond to the mean and standard deviation for quantitative variables and to the number and frequency for qualitative variables; ^2^ *p*-value obtained by ANOVA (*p* < 0.05) for quantitative variables and by X^2^ test (*p* < 0.05) for qualitative variables.

**Table 5:** Blood gas analysis in arterial and venous umbilical cord blood at delivery as a function of occupational exposure.

	No Exposure		Chemical/Biological Risk		Physical Risk		*p*-Value ^2^
	Mean o fr ^1^	±SD o % ^1^	Mean o fr ^1^	±SD o % ^1^	Mean o fr ^1^	±SD o % ^1^	
arterial PO2 (mmHg)	7.28	±0.11	7.28	±0.12	7.29	±0.09	0.778
	50.53	±9.77	52.16	±10.69	50.95	±9.71	0.404
	24.83	±17.57	21.96	±10.18	20.46	±8.70	**0.027**
Venous pH	7.33	±0.11	7.31	±0.11	7.33	±0.57	0.622
Venous PCO2 (mmHg)	42.69	±8.04	44.04	±9.38	43.06	±7.28	0.398
PO2 venoso (mmHg)	29.01	±15.61	28.17	±13.45	25.53	±7.26	0.086

^1^ Values correspond to the mean and standard deviation for quantitative variables and to the number and frequency for qualitative variables; ^2^ *p*-value obtained by ANOVA (*p* < 0.05) for quantitative variables and by X^2^ test (*p* < 0.05) for qualitative variables.

**Table 6:** Association between maternal and newborn factors and occupational exposure during pregnancy.

	Chemical/Biological Risk		Physical Risk	
	OR_c_ ^1^ 95%CI ^2^	OR_a_^3^ 95%CI ^2^	OR_c_ ^1^ 95%CI ^2^	OR_a_^3^ 95%CI ^2^
**Abortions**	1.26 [0.27–5.77]	1.45 [0.29–5.93]	1.44 [0.34–6.23]	1.17 [0.22–6.10]
**PO_2 arterial_**	0.94 [0.46–1.89]	0.18 [0.02–1.49]	1.68 [0.87–3.27]	1.96 [0.88–4.08]
**Apgar 1 min**	0.78 [0.33–1.83]	1.35 [0.31–5.85]	0.44 [0.20–0.97]	0.35 [0.13–0.89]

^1^ Crude odds ratio; ^2^ 95% confidence interval; ^3^ adjusted odds ratio.

## Data Availability

No new data were created or analyzed in this study. Data sharing is not applicable to this article.
